# Functional Genomics Complements Quantitative Genetics in Identifying Disease-Gene Associations

**DOI:** 10.1371/journal.pcbi.1000991

**Published:** 2010-11-11

**Authors:** Yuanfang Guan, Cheryl L. Ackert-Bicknell, Braden Kell, Olga G. Troyanskaya, Matthew A. Hibbs

**Affiliations:** 1 Department of Molecular Biology, Princeton University, Princeton, New Jersey, United States of America; 2 Lewis-Sigler Institute for Integrative Genomics, Princeton University, Princeton, New Jersey, United States of America; 3 The Jackson Laboratory, Bar Harbor, Maine, United States of America; 4 Department of Computer Science, Princeton University, Princeton, New Jersey, United States of America; University of Illinois at Urbana-Champaign, United States of America

## Abstract

An ultimate goal of genetic research is to understand the connection between genotype and phenotype in order to improve the diagnosis and treatment of diseases. The quantitative genetics field has developed a suite of statistical methods to associate genetic loci with diseases and phenotypes, including quantitative trait loci (QTL) linkage mapping and genome-wide association studies (GWAS). However, each of these approaches have technical and biological shortcomings. For example, the amount of heritable variation explained by GWAS is often surprisingly small and the resolution of many QTL linkage mapping studies is poor. The predictive power and interpretation of QTL and GWAS results are consequently limited. In this study, we propose a complementary approach to quantitative genetics by interrogating the vast amount of high-throughput genomic data in model organisms to functionally associate genes with phenotypes and diseases. Our algorithm combines the genome-wide functional relationship network for the laboratory mouse and a state-of-the-art machine learning method. We demonstrate the superior accuracy of this algorithm through predicting genes associated with each of 1157 diverse phenotype ontology terms. Comparison between our prediction results and a meta-analysis of quantitative genetic studies reveals both overlapping candidates and distinct, accurate predictions uniquely identified by our approach. Focusing on bone mineral density (BMD), a phenotype related to osteoporotic fracture, we experimentally validated two of our novel predictions (not observed in any previous GWAS/QTL studies) and found significant bone density defects for both *Timp2* and *Abcg8* deficient mice. Our results suggest that the integration of functional genomics data into networks, which itself is informative of protein function and interactions, can successfully be utilized as a complementary approach to quantitative genetics to predict disease risks. All supplementary material is available at http://cbfg.jax.org/phenotype.

## Author Summary

Many recent efforts to understand the genetic origins of complex diseases utilize statistical approaches to analyze phenotypic traits measured in genetically well-characterized populations. While these quantitative genetics methods are powerful, their success is limited by sampling biases and other confounding factors, and the biological interpretation of results can be challenging since these methods are not based on any functional information for candidate loci. On the other hand, the functional genomics field has greatly expanded in past years, both in terms of experimental approaches and analytical algorithms. However, functional approaches have been applied to understanding phenotypes in only the most basic ways. In this study, we demonstrate that functional genomics can complement traditional quantitative genetics by analytically extracting protein function information from large collections of high throughput data, which can then be used to predict genotype-phenotype associations. We applied our prediction methodology to the laboratory mouse, and we experimentally confirmed a role in osteoporosis for two of our predictions that were not candidates from any previous quantitative genetics study. The ability of our approach to produce accurate and unique predictions implies that functional genomics can complement quantitative genetics and can help address previous limitations in identifying disease genes.

## Introduction

Understanding the genetic bases of human disease has been an overarching goal of biology since the foundation of genetics as a scientific discipline. Efforts in quantitative genetics have utilized new laboratory technology to quickly genotype and phenotype large populations in order to determine which sequence features are most related to specific phenotypes. There are currently two major quantitative genetics approaches used to identify these genotype-phenotype associations [1]. First, linkage mapping examines genetically well-characterized populations, such as the progeny of the crosses of reference strains or individuals related through a known pedigree, to identify quantitative trait loci (QTL) that contain causal mutations. Second, genome-wide association studies (GWAS) can be performed on a more arbitrary population to identify common genetic factors associated with a phenotype. Hundreds of GWAS and QTL studies have been performed in humans and in model organisms, resulting in the identification of thousands of loci associated with phenotypes and diseases.

Despite promising results, each of these approaches for quantitative genetics have common and unique unresolved issues that limits their utility. Both QTL and GWAS approaches can suffer from sampling biases. Population structure and proper selection of representative case and control groups are challenges for many GWAS, while linkage disequilibrium and limited genetic diversity are challenges for many QTL studies [1]–[4]. Further, many linkage mapping QTL studies lack the statistical power to narrowly define a causal loci, often resulting in regions spanning entire chromosomes that contain hundreds of candidate genes [5]. While these QTL regions are often broad, they can typically explain a large fraction of phenotypic variation. In contrast, GWAS typically define narrow regions of interest, but the amount of heritable variation explained by these loci tends to be small, possibly due to epistatic effects, rare alleles, or sampling biases [6]. For example, a meta-analysis GWAS of bone mineral density (BMD) based on nearly 20,000 genotyped and phenotyped individuals can only account for less than 3% of the observed heritability of BMD [7].

Thus, there is a strong need for complementary approaches to quantitative genetic techniques that are independent from the biases inherent in linkage mapping QTLs and GWAS. Many of the shortcomings of quantitative genetics could be attenuated by considering the functional roles of proteins and by evaluating existing large-scale experimental evidence. As such, we propose a complementary, alternative approach to discovering gene-phenotype associations that applies machine learning techniques to functional genomic measurements of the activities of genes and proteins (*e.g.* expression, interactions, etc.) to identify candidate genes that may be involved in a phenotypic outcome.

Recent efforts have undertaken the task of summarizing the entirety of the experimental genomic literature into functional networks of genes. These networks typically encode genes as nodes, and contain experimental evidence of the relationships between genes as edges between nodes. Computationally exploring diverse functional genomics data through networks has been intensively studied, with the purpose of elucidating the functional roles of genes [8]–[11], predicting physical interactions [12], [13], and determining pathway structures [14]. Functional networks have been produced in several organisms, including yeast [9], [15], worm [11], [16], plant [17], mouse [10], and human [18]. These networks have the advantage of being able to efficiently handle very-high dimensional data and allow for visual analysis of results. However, attempts to extract phenotypic information from these functional networks are limited, with only the most naïve summarization of links being applied in model organisms [11], [17], [19] as well as human [20]. In order to utilize functional networks to identify phenotypically important genes in higher organisms, where we face complex biology and increased data heterogeneity, more sophisticated computational approaches must be developed.

Historically, the fields of quantitative genetics and functional genomics have been largely isolated from each other. A major exception to this observation is the recent development of genetical genomics and expression QTL studies [21], [22]. These approaches use the mapping populations of traditional quantitative genetics, but utilize gene expression measurements as the phenotype to map, rather than direct physiological or disease phenotypes. These studies have begun to illuminate the regulatory programs of gene networks and have quantified the effects of genetic diversity on gene expression. However, these efforts suffer from the same technical problems as other QTL studies, and the interpretation of results is currently limited and is often disconnected from more clinically relevant phenotypes and analyses [23].

We propose that functional genomics approaches can complement the potential shortcomings of quantitative genetics results in two ways: first, by identifying candidates that may have been missed due to biases in sampling or low allele frequency; and second, by prioritizing candidates in loci containing many genes due to limited mapping resolution. Here, we adopt a state-of-the-art machine learning algorithm (support vector machine) to analyze the functional network of the laboratory mouse to identify genes involved in phenotypes and diseases. We show that our approach significantly out-performs previous naïve functional genomic methods used to predict phenotypes. Further, we demonstrate that our results are complimentary to quantitative genetics methods since a statistically significant number of our predictions fall within QTLs or GWAS loci, but several of our most confident predictions fall outside of these regions as well. We have experimentally validated a phenotypic role for two genes predicted to be involved in bone mineral density (BMD), a risk factor for osteoporotic fracture, which were not identified by any previous quantitative genetics study (*Timp2* and *Abcg8*). Our results concretely demonstrate that the combination of quantitative genetics and functional genomics approaches can more comprehensively associate genes with phenotypes or diseases, which may aid in identifying risk factors and potential drug targets.

## Results/Discussion

In this study, we develop a new algorithm that accurately predicts the phenotypic effects of genetic perturbations based on functional genomic data, and we demonstrate that this approach complements the results of quantitative genetics studies. In the following sections, we first demonstrate that combining a genome-wide functional relationship network with a state-of-the-art machine learning algorithm can produce improved predictions of genotype-phenotype relationships compared to previous naïve algorithms. Second, we observe that both existing prior knowledge and the biological nature of phenotypes affect the accuracy of our approach. Third, we quantitatively show that our results compensate for some of the shortcomings of prior GWAS and QTL studies by providing complementary predictions based on large-scale functional genomic data. Finally, we experimentally demonstrate the power of this alternative approach by *in vivo* validation of two of our unique predictions of genes involved in bone physiology. The predictions made by our approach for all examined phenotypes (as well as input data files, raw data outputs, and source code) are available online at http://cbfg.jax.org/phenotype.

### Machine learning classification based on a functional relationship network accurately predicts gene-phenotype associations

**Figure 1:**
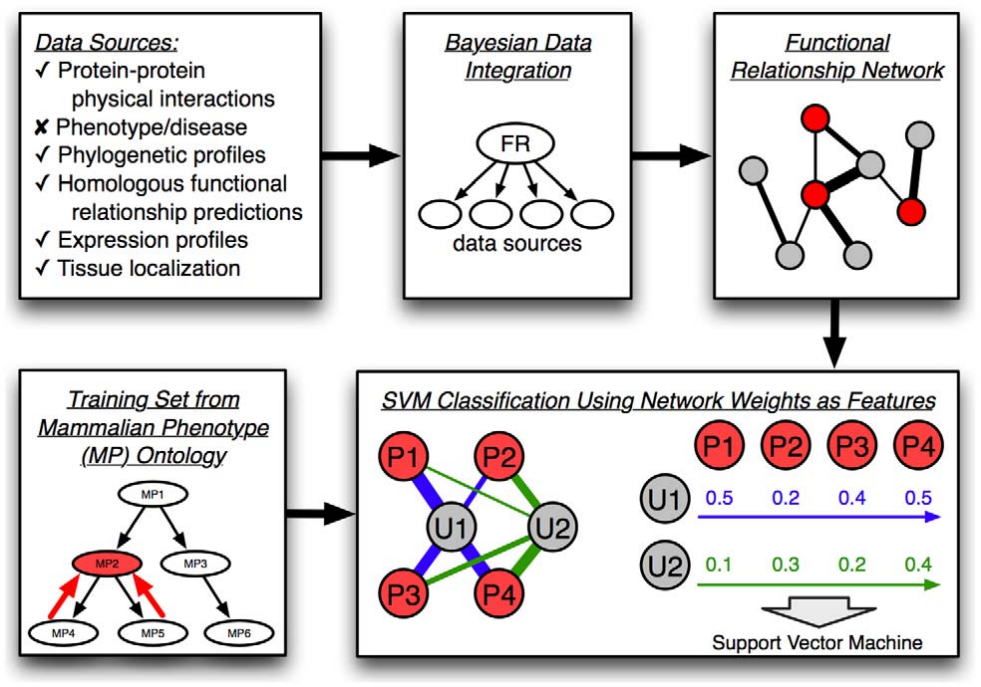
Schematic of our SVM-based functional genomics approach to phenotype prediction. As shown in the upper half, a functional relationship network was first constructed for the laboratory mouse based on integration of diverse data types, excluding phenotype and disease data to avoid contamination in evaluation. These data were integrated together using Gene Ontology annotations as a gold standard using an established Bayesian pipeline [10]. The resulting network consists of genes as nodes and connections between them representing the probability of two genes participating in the same biological process. This network was used as the basis for an SVM classifier to predict genes associated with phenotypes. As shown in the lower half, annotations to the mammalian phenotype (MP) ontology were used to create gold standards (i.e. training sets) for each SVM. Annotations were propagated along the ontology tree to produce positive training examples (genes associated with the phenotype, labeled P and shown in red). All other genes were considered as unknowns (labeled U and shown in grey). SVMs were provided with input features consisting of network connection weights to positive genes for each phenotype and each SVM was trained to classify unknown examples in this phenotype-specific feature space.

Integrated functional relationship networks have the advantage of summarizing multiple complex datasets into a concise, visually interpretable graph representation where genes are nodes and connections between them represent the probability that two genes work together [8]–[11], [15], [17]–[19]. We applied a Bayesian network approach for supervised data integration, which assesses the conditional probability that individual data sources contain evidence for gene relationships based on a training set of positive examples (*e.g.* gene pairs known to interact) and negative examples (*e.g.* gene pairs not known to interact). Given these learned conditional probabilities for each data source and prior probabilities for gene relationships, Bayesian inference is used to generate a network defined by pair-wise posterior probabilities of functional relationships between all genes. In order to use this network to predict genotype-phenotype associations, we must exclude phenotypic data to avoid circularity. Therefore we gathered diverse genomic data for inputs, including protein-protein physical interactions [24]–[27], phylogenetic profiles [28], [29], homologous functional relationship predictions in yeast [9], and expression and tissue localization data [30]–[32] to construct a functional relationship network for the laboratory mouse (Figure 1; input data sources and integration methods are available in Supplemental Text S1). We integrated these diverse data using a Bayesian network trained using a gold standard derived from the Mouse Genome Informatics (MGI) [33] annotations to the Gene Ontology (GO) [34] biological process branch as described previously [10] and in Supplemental Text S1. This procedure resulted in a probabilistic functional relationship network for the laboratory mouse that combines diverse knowledge and data.

Given this integrated functional relationship network, we applied two methodologies to generate hypotheses about genotype-phenotype associations. Each method is supervised, and thus requires a starting set of genes known to be associated with each phenotype or disease of interest. Here, we used the mammalian phenotype (MP) ontology [35] annotations to create our training sets of gene-phenotype associations. For each phenotype examined, genes were considered positive examples if any allele is annotated to the phenotype and all other genes were considered negative examples.

First, our new approach treats the connection weights from the integrated network (*i.e.* the inferred probabilities that genes are functionally related) as features for support vector machine (SVM) classification [36]. In order to reduce the size of the feature space, only connection weights to known genes associated with a phenotype (*i.e.* positive examples) are used for training. We then apply the trained SVM to classify all genes for each phenotype (Figure 1). For ease of interpretation, the raw SVM scores were normalized to represent probabilities of gene-phenotype association as described in the Methods section. The result of this approach is used in all of our later analyses, and is denoted simply as “SVM” below.

Second, in order to establish a baseline for comparison, we also explored a naïve method previously used in other model organisms [11], [17], [19], as well as human [20], which assigns a score to an unknown gene by its summed connection weights to all known genes associated with a phenotype in the integrated network. For this method, genes were ranked according to their summed connection weight, which we denote as “summed weight” throughout the text. (In addition to these two approaches, we also trained SVMs directly on the input data used to create the functional relationship network. As detailed in Supplemental Text S2, this approach induced a dramatic increase in feature space and running time, and was always outperformed by our method.)

**Figure 2:**
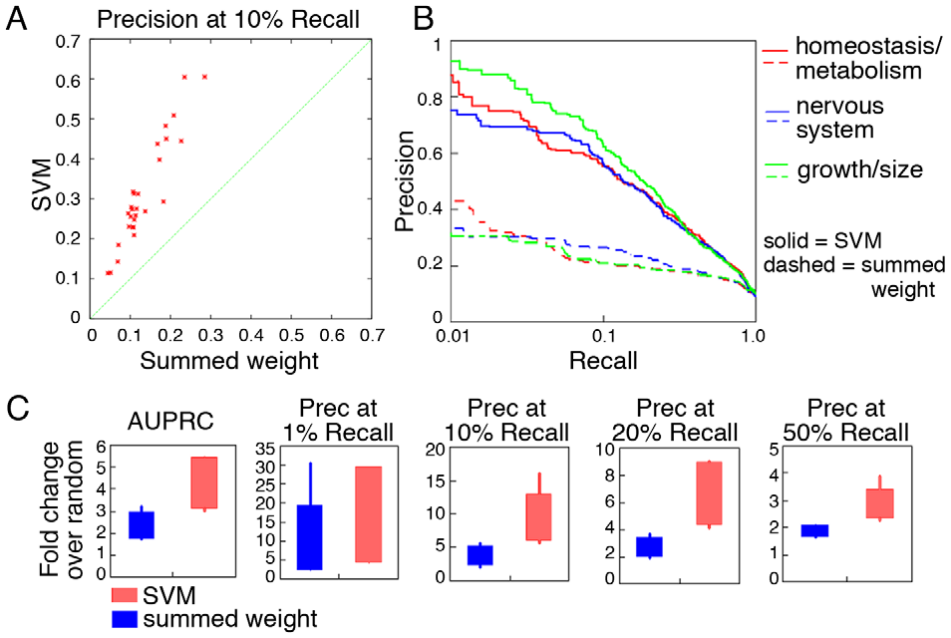
Significantly improved performance of our network-based SVM method for predicting well-defined phenotypes. Thirty well defined, high level MP terms were obtained from MGI [37], which represent a wide sampling of phenotypes. A. Precision at 10% recall is shown for these 30 MP terms using our method (SVM; y-axis) and the summed weight method (x-axis). B. Precision-recall curves for an indicative selection of three high level phenotypes for our SVM-based method and the summed weight method. Full results for all examined phenotypes are available at the supplementary website (http://cbfg.jax.org/phenotype) and in Supplemental Figure S1. C. Box and whisker plots comparing our SVM approach to summed weight using several different summary statistics, including AUPRC (area under the precision recall curve), precision at 1%, 10%, 20% and 50% recall, each expressed as fold change over random precision. In all cases our approach outperforms the summed weight method.

Each of these approaches was applied to a set of 1157 diverse phenotypes defined by the mammalian phenotype (MP) ontology [35]. Predictions were computationally evaluated through bootstrapping for each method and phenotype. Performance summary statistics were calculated, including the area under the precision-recall curve (AUPRC) and precision at *n*% recall. To establish general performance measures, we first focused on 30 MP terms from the first level of the ontology, which represent a wide sampling of “high-level,” well-characterized phenotypic areas [37]. Cross-validation performance for these 30 high-level terms revealed significant improvement in performance for our new SVM-based approach compared to the summed weight method (Figure 2). The median AUPRC for these terms using SVM is roughly 1.8 fold greater than for the summed weight approach (results for a sampling of three representative MP terms are shown Figure 2B; full results for all 30 phenotypes are available in Supplemental Figure S1). This performance improvement is especially apparent for our most confident predictions (*i.e.* at the low-recall, high-precision end), which is most important for subsequent biological validation where only a handful of candidates can be reasonably examined. At 1% recall (roughly 200 predictions), our SVM approach achieved a median of 75% precision, compared to 43% for the summed weight method; and at 10% recall (roughly 2000 predictions) our SVM approach outperforms summed weight by 40% to 15%. The comparisons of precisions at multiple levels of recall confirm the overall improved quality of our algorithm over naïve methods (Figure 2C).

### SVM-based phenotype predictions are most accurate for large phenotype terms

**Figure 3:**
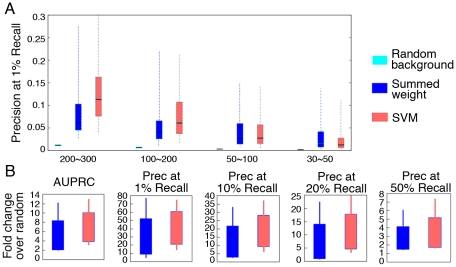
Network-based SVM approach performs best for reasonably large terms. A. Phenotypes were grouped by the number of genes annotated to each MP term (200∼300, 100∼200, 50∼100 and 30∼50, respectively). The performance of summed weight (dark blue), and our network-based SVM approach (red) are compared against random (cyan) using box-and-whisker plots of precision at 1% recall. B. Fold improvement of summary statistics over random for our network-based SVM approach (red) and the summed weight method (dark blue) for reasonably large terms (>1.5% of the genome annotated, *i.e.* 200–300 genes).

Prediction algorithms often show drastic differences in baseline performance related to the number of training examples (*i.e.* the number of genes annotated to each MP term) [38], which is an important factor in fully evaluating the strength of algorithms. We therefore assembled all phenotype terms into groups of 30–50, 50–100, 100–200, and 200–300 annotated genes to assess the impact of term size on results. Both the summed weight and the SVM method achieved better performance than random, regardless of term size (Figure 3A). However, our SVM-based method demonstrated a more significant improvement for reasonably large terms (with more than 100 genes annotated, or >0.5% of the genome; shown in Figure 3B). For example, in the 200–300 annotation group, our SVM approach achieved an average improvement of 1.78 fold over summed weight, and in the 100–200 annotation group we observed a 1.67 fold improvement. The superior performance of our SVM-based method implies that more sophisticated machine learning techniques are better able to fully extract phenotypic information from functional networks than previous simpler approaches.

### Phenotype prediction is particularly accurate for biologically specific phenotypes

As discussed above, there is a significant effect on prediction accuracy based on the number of known gene annotations to each phenotype. In addition to this size effect, there appears to be a strong correlation between prediction accuracy and the ability of a phenotype to be accurately and reproducibly measured. For example, among the phenotypes most accurately predicted by our approach are “decreased IgE level” (MP:0002492) and “decreased circulating free fatty acid level” (MP:0002702). Each of these is a concrete phenotype that is measurable using an unbiased metric such as “concentration” or “width”. In contrast, among the most poorly predicted phenotypes are “head bobbing” (MP:0001410), “disheveled coat” (MP:0001511), and “lethargy” (MP:0005202), which are more qualitative in nature (*i.e.* presence/absence calls) or which are measured based on a subjective “severity score”.

While it is difficult to concretely assess the notion of “phenotypic specificity,” we generally observe in our overall results that more concrete phenotypes tend to perform more accurately. In order to quantify this effect, we conducted a small, blinded survey of 19 laboratory biologists. Survey respondents ranked phenotypes on a scale from 1 (not specific, qualitative) to 5 (highly specific, quantitative). Based on these results, we found a small, but significant, difference between the top and bottom 20 phenotypes ranked by overall precision (3.6 versus 3.3; *p* = 0.008; full survey results in online supplement), which confirms our observation that more quantitative phenotypes tend to perform better. This phenomenon is not surprising in that functional genomic evidence is more likely to be informative for well-defined phenotypes. However, this effect is also promising in that better defined phenotypes are more likely to reflect specific molecular-level changes that may be more relevant from a drug target or clinical diagnosis perspective.

### Network-based SVM predictions complement quantitative genetics in identifying disease genes

Our prediction approach is based on an integrated functional relationship network, rather than pure genotype and phenotype information, and thus potentially avoids several of the caveats of quantitative genetics methods. While we expect that our approach and GWAS/QTL studies will share many candidates, since the underlying assumptions of functional genomics and quantitative genetics are very different, we also expect to obtain predictions unique to each method. We evaluated the utility of our functional genomic approach both by comparing our predictions to previous quantitative genetics loci, and by experimentally validating predictions unique to our approach.

**Figure 4:**
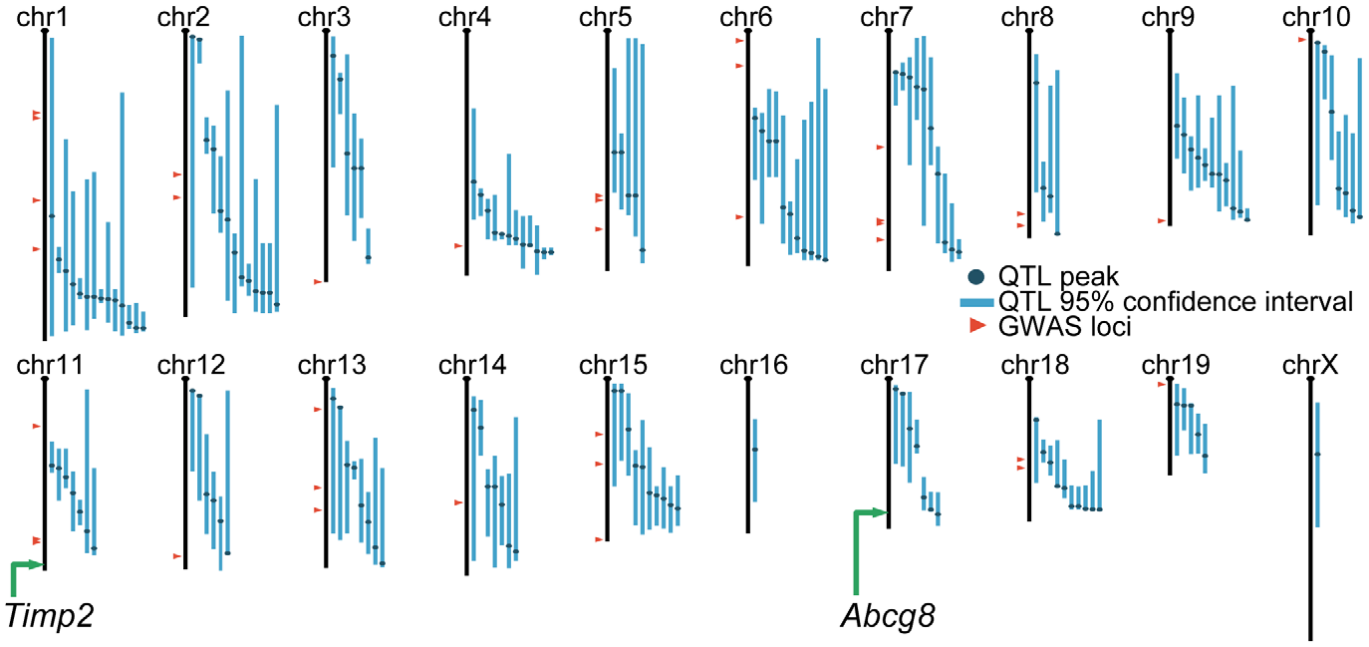
Two of our accurate predictions were not implicated by previous quantitative genetics studies. A chromosomal map of the laboratory mouse genome is shown, labeled with results compiled from previous human GWAS (red triangles) and mouse QTL studies (blue lines) of bone mineral density (BMD) [5]. Due to the limited resolution of some QTL studies, 83% of the mouse genome lies underneath the 95% confidence interval of at least one QTL. We experimentally confirmed two of our most confident predictions for bone defects that were not implicated in any previous QTL or GWAS effort: *Timp2* and *Abcg8* (green arrows). While *Abcg8* falls underneath a single QTL on chromosome 17, it was not a candidate gene for the loci as there are no polymorphisms within 20kb of *Abcg8* in the strains crossed for this study [39]. *Timp2* does not lie under any previously implicated loci.

We selected bone mineral density (BMD) as an example to evaluate our approach since this is an extensively studied heritable trait in both human populations and mammalian model organisms [5]. We compared the predictions for the phenotype “abnormal bone mineralization” (MP:0002896) from our functional genomic approach against a comprehensive list of mouse linkage QTLs and human GWAS results examining BMD [5]. Due to the limited resolution of some mouse QTL studies, fully 83% of mouse genes lie under the confidence interval of at least one BMD QTL reported in the literature (Figure 4). Despite this lack of specificity, we still observe a significant overlap between our top predictions and these QTLs as 93 of our top 100 predictions are contained within a QTL confidence region (hypergeometric *p*-value = 0.002). Similarly, genomic regions within 5cM of a QTL peak contain 16% of mouse genes, but contain 71 of our top 100 predictions (hypergeometric *p*-value = 7×10^−32^).

While this overlap is significant, a large number of our most confident predictions (∼30% of the top 100) do not fall within 5cM of a QTL peak and are thus not likely candidates from previous studies. We consider these to be candidates likely missed by prior quantitative genetics studies due to sampling, population biases, epistasis, or other circumstances. In fact, two of our most confident predictions for genes associated with BMD are not candidates from previous quantitative genetics studies, but both have been experimentally verified as described below.

### *In vivo* testing validates a role for *Timp2* and *Abcg8* in mammalian bone density

For experimental validation, we selected two of our genes predicted for association with BMD that are not candidates from any previously reported linkage QTL or GWAS regions [5]: *Timp2* and *Abcg8*. These genes are our two most confident predictions for involvement in BMD that are not quantitative genetics candidates and that have existing, live knockout strains available for testing (see Supplemental Table S3 for all top BMD predictions). In the laboratory mouse, the *Timp2* gene falls on the distal end of chromosome (Chr) 11, outside of all previous QTLs identified on Chr 11. The *Abcg8* gene lies on the distal end of Chr 17. One previous QTL study found a BMD QTL on Chr 17 that contains over 100 genes, including *Abcg8* [39]. However, *Abcg8* was not considered as a possible candidate gene in this study because there is no known polymorphism within 20 kb of *Abcg8* in the two strains crossed for linkage mapping (NZB/B1NJ and RF/J). None of the 20 BMD loci identified in human GWAS lie near either *Timp2* or *Abcg8* (Figure 4).

**Figure 5:**
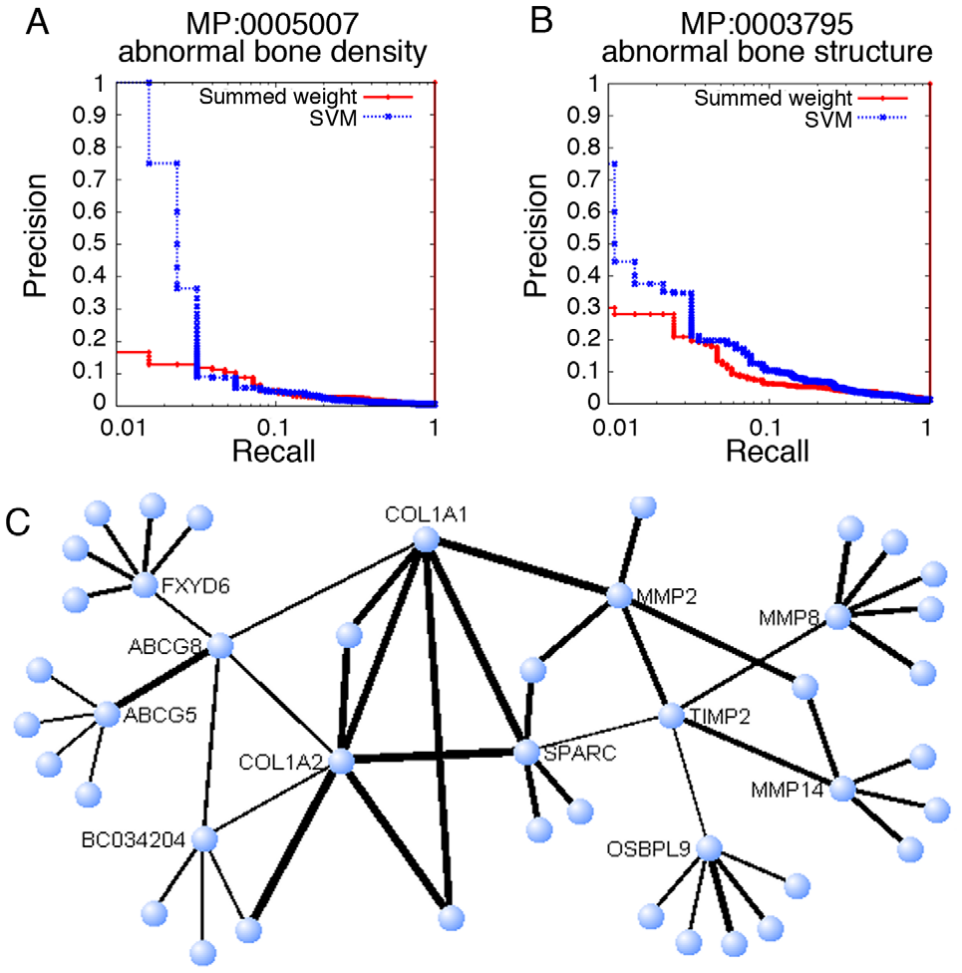
Predicting novel candidates associated with bone phenotypes. Precision-recall curves of the cross-validation results for A. “abnormal bone density” (MP:0005007) and B. “abnormal bone structure” (MP:0003795). For both phenotypes, our network-based SVM achieved a better performance than summed weight. C. A network view of functional relationships centered around *Timp2* and *Abcg8*. Nodes indicate genes, and the thickness of lines indicates relationship confidence. Both *Timp2* and *Abcg8* are connected to several genes that demonstrate a bone mineralization phenotype or are otherwise implicated in osteoporosis. Networks were visualized using VisANT [50].

We have high confidence in the accuracy of these candidates for two reasons. First, our approach produced accurate cross-validation results for many osteoporosis and BMD related phenotypes such as “abnormal bone density” (MP:0005007) and “abnormal bone structure” (MP:0003795) (Figure 5A–B). Second, in our functional network, *Timp2* and *Abcg8* are linked to several genes known to be related to BMD and bone diseases in the Online Mendelian Inheritance in Man (OMIM) database (Figure 5C and Supplemental Tables S1 and S2). For example, *Timp2* is directly linked to *Mmp2*, which is a known player in hereditary osteolysis (OMIM # 259600). It is also linked to the metalloproteinases *Mmp8* and *Mmp14*, the collagen *Col1a1*, and the glycoprotein *Sparc*, all of which have been associated with bone defects in the literature [40], [41]. Among the top interactors of *Abcg8* are the collagens *Col1a2* and *Col1a1*, which are involved in osteoporosis (OMIM #166710). Additional interactors of *Abcg8* include the bone related genes *Sparc* and the proteoglycan *Bgn*. All of these connections are supported by multiple evidence sources, including expression, physical interactions and phylogenetic profiles (Supplemental Table S1).

Despite the connections apparent in our functional network, to our knowledge no bone related phenotypes have been reported for either *Timp2* or *Abcg8* knockout mouse strains. However, there is limited evidence in the literature that these genes may play a role in bone biology. Polymorphisms in the human *Timp2* gene were found to be weakly associated with increased risk of non-vertebral osteoporotic fracture in a small study of post-menopausal women [42]. *Abcg8* is involved in cholesterol absorption and serum cholesterol levels [43], which are processes that have been related to bone homeostasis through other genes [44], [45]. These two cases show that our integrative approach is able to draw implicit information from a variety of high throughput data to confidently associate these two proteins with BMD defects.

**Figure 6:**
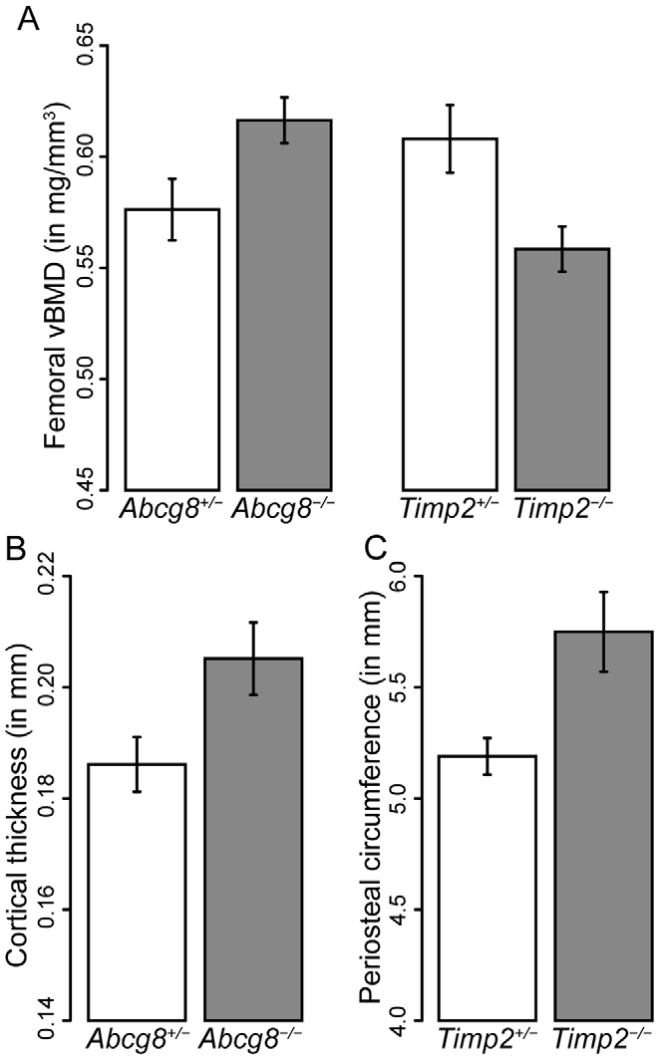
Bone phenotype assessment for *Timp2* and *Abcg8* deficient mice. A. Total femoral volumetric bone mineral density (vBMD) is significantly lower in *Timp2* deficient male mice (*N* = 5) as compared to haploinsufficent littermate controls (*N* = 7, *p* = 0.033). In contrast, a significant increase in vBMD was observed in *Abcg8* deficient male mice (*N* = 7) as compared to haploinsufficent littermate controls (*N* = 9, *p* = 0.044). Error bars represent standard error. In addition to BMD effects, morphological defects were observed in both strains. B. *Abcg8* deficient male mice show an increase in cortical thickness compared to controls (*p* = 0.032). C. *Timp2* deficient male mice exhibit increased periosteal circumference compared to controls (*p* = 0.0105).

We examined femoral volumetric BMD (vBMD) in male *Timp2* and *Abcg8* knockout mice at 16 weeks of age. Animals homozygous for deletion were compared to heterozygous littermate controls. As shown in Figure 6A, we observed a significant decrease of roughly 8% of vBMD in *Timp2* ^−/−^ male mice (*p*-value = 0.033) and a significant increase of roughly 6.5% was observed in *Abcg8^−/−^* male mice (*p*-value = 0.044). Most individual mouse QTLs account for a 3–6% change in vBMD [5], which places our observed differences in the high end of this range. We also examined phenotype results from the Deltagen and Lexicon collections of over 200 knockout mouse strains (http://www.informatics.jax.org/external/ko/) to assess the likelihood of identifying genes with a significant affect on BMD. While these strains were not randomly selected, we can use these results to gain an estimate of how often single gene deletions affect bone density. Of 206 strains tested, only 20 exhibited a significant alteration in BMD, indicating a roughly 10% background rate. Thus, our confirmation of 2 out of 2 predictions is well above the expected result by chance.

Furthermore, we observed morphological defects in the bones of both strains, including an increase in periosteal circumference in the *Timp2* ^−/−^ mice (*p*-value = 0.0105) and an increase in cortical thickness in the *Abcg8* ^−/−^ mice (*p*-value = 0.032) as shown in Figure 6BC. Osteoporotic fracture risk increases with decreasing bone mass, but morphologic factors such as bone shape also contribute to fracture risk [46]. The decrease in bone density seen in *Timp2^−/−^* and the increase density seen in *Abcg8^−/−^*, along with the noted differences in bone morphology, indicate that these genes are likely related to osteoporotic fractures. Neither of these genes is a candidate from any previous quantitative genetics study of BMD, which indicates that our approach produces results that are complementary to GWAS and QTL studies.

### Conclusions

We have developed a novel SVM-based classification method to predict genotype-phenotype associations based on a probabilistic functional relationship network integrated from diverse data sources. Through bootstrapping and cross validation we confirmed its superior performance compared to previous approaches. Using osteoporosis related phenotypes as an example, we have computationally demonstrated and experimentally validated how integration of functional genomics data can facilitate disease gene identification in a complementary manner to quantitative genetics approaches.

This study demonstrates the potential for integrative functional relationship networks to be applied in new ways, especially when successfully combined with sophisticated machine learning techniques. Functional networks have been intensively studied during recent years, resulting in multiple networks available for several model organisms [8]–[11], [15]–[19] as well as for humans [18], [20]. In addition to the original applications of these networks, our results demonstrate that functional relationship networks can be used to accurately predict gene-phenotype associations, and that supervised machine learning approaches outperform the simple fusion methods previously applied to this problem [11], [17], [19], [20]. By combining two computational learning methodologies (Bayesian network integration of diverse data into a probabilistic relationship network and SVM classification), we were able to utilize the complementary advantages of each method to produce accurate results. We anticipate that such an approach could be a prototype for other forms of network-assisted prediction methods in cases where gold standard positive and negative examples are available for training.

We have also shown that our integration of functional genomics data is able to identify potential disease genes not yet identified by any quantitative genetics screens. The caveats of quantitative genetics, including sampling biases, rare allele effects, epistasis, and potentially limited explanatory power have been recognized for years [1]–[4]. Our approach suggests a complementary new avenue to address some of these limitations through the analysis of existing genomics data in model organisms, which is generally not included in quantitative genetics studies. We expect that a combination of complementary approaches will be required to realize the ultimate goals of improved genetic diagnosis, treatment, and prevention that form the basis of personalized medicine.

## Methods

### Constructing the functional relationship network

To avoid circularity in the phenotype prediction process, we created a functional relationship network that excludes phenotypic data. We pre-computed this network following the Bayesian method described in [10] by integrating diverse sources of genome-scale data. This results in a network with genes as nodes and links between them representing the probability that the pair participates in the same biological processes. The input data used to generate this network is listed in Supplemental Text S1 and the complete network is downloadable at http://cbfg.jax.org/phenotype.

### Gold standards for phenotype predictions

We utilized the annotations to the mammalian phenotype (MP) ontology [35] as the gold standard gene sets. The MP ontology is organized into a multi-level hierarchy, with broader terms describing more general phenotypes at the top and more specific terms describing detailed phenotypes toward the bottom. Within this hierarchy, any annotation to a child node implies annotation to all of its parent nodes. Therefore, for each MP term, positive examples were taken as the genes annotated directly to this term or to any descendent of this term. Negative examples were assumed to be all other genes.

We obtained the phenotype annotations for mouse from MGI [33] in Jan 2009. These included 134722 entries, containing alleles for 11382 genes in total (∼55% of the mouse proteome). If any allele of a gene was annotated to a phenotype, we associated that gene with the phenotype. For each term, the number of positive examples *n_p_*, corresponds to all genes known to be associated with the phenotype or its descendents in the MP hierarchy. All other genes were considered as negative examples; the total number of negative examples is denoted as *n_n_*.

### Phenotype prediction by summed weight

A summed weight approach has been applied in previous studies [11], [17], [19], [20] to predict phenotypes of uncharacterized mutants in other model organisms. Variants of this approach have been used for function prediction and other forms of analysis [18]. For each phenotype, a score, *f*, is calculated for each gene, *x*, as the sum of all links between the gene and all positive examples from the gold standard:
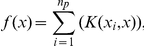
where *K(x_i_, x)* represents the connection weight between the genes *x_i_* and *x* in the probabilistic functional network.

### Phenotype prediction using positive examples as features in a SVM

Predicting phenotypic effects by summing connection weights to positive examples has achieved satisfactory performance [11], [17], [19], [20]. Although straightforward, this approach does not fully explore the predictive potential of functional networks that can be achieved by applying more principled machine learning techniques. We therefore designed a new method combining Support Vector Machine (SVM) classifiers with a functional relationship network to predict phenotypes associated with each mouse gene.

For each phenotype, we constructed a specific feature space consisting of the network connection weights to all positive examples of the phenotype. Therefore the number of features varies across different phenotypes and is equal to *n_p_*, the number of genes positively associated with that phenotype. These features were used as input vectors for a set of linear SVMs [36]:
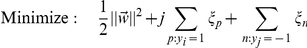


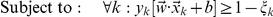
where *x_i_* is the feature vector for gene *i* (*i.e.* the connection weight to the positive examples), *y_i_* equals to 1 or −1 depending on whether gene *i* is annotated to the phenotype term or not, *p* is any gene annotated to the term in study, and *n* is any of the other genes.

Alternately, we could use the connection weight to all examples as the input vectors, regardless of whether they are positive or negative examples. However, this construction is excessively time-consuming for SVM calculations. For example, in a Lagrangian SVM, the running time is approximately *O(nN^2^)*, where *n* is the number of examples and *N* is the number of features. In comparison, our approach takes only an average of 6 minutes (approximately 100 fold reduction in time) for a 25-round bootstrap validation to generate the prediction results for each phenotype term. Another intuitive algorithm is to directly input all original data into a linear SVM [38]. However, this approach is both time consuming due to the large number of features involved and performed less accurately than our approach (see Supplementary Text S2).

### Predicting phenotypes and evaluating predictive performance by bootstrapping

In order to limit over-fitting, and because each phenotype is only associated with a limited set of genes, we consider bootstrap cross-validation to be an ideal method for estimating error rates [47]. We therefore applied bootstrap aggregation to predict genes associated with each phenotype and to estimate accuracy. Intuitively, this method trains models on a subset of genes and tests it on a different subset of genes repeatedly, thus minimizing the possibility of over-fitting and the effects of potentially mis-annotated genes. Specifically, for each iteration, genes were sampled with replacement to form a training set, and all the remaining genes form a test set. Classification values were only recorded for the test set during each iteration. The final outputs were calculated as the median of the out-of-bag values across 25 independent bootstraps, and the precision-recall curves were derived from these median values.

### Probability estimation

The outputs from the SVMs represent the distances from the examples to the separating hyperplane [36], which are not intuitive to understand. To make the value of these outputs more comprehensible, we estimated the probability of being annotated to a phenotype by fitting the output distribution of positive and negative examples with two normal distributions. According to Bayes' rule,
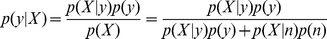
where
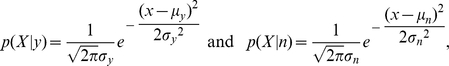
where *X* is the raw output value for a gene, *y* represents positive examples, *n* represents negative examples, *σ_y_* and *σ_n_* are the standard deviations of the raw outputs for positive and negative examples, and *μ_y_* and *μ_n_* are the mean of the raw outputs for positive and negative examples. Based on these distributions we estimate the probability of a gene being associated with a phenotype as *p(y|X)*, given its observed value *X*. Outputs with a value lower than the average of negative examples are assigned as zero. Transforming to probability does not affect the ordering of results, and consequently has no effect on our performance evaluation metrics. We provide the complete list of predictions in terms of probability on our supporting website http://cbfg.jax.org/phenotype.

### Performance evaluation

To assess the performance of the phenotype predictions, we obtained precision-recall curves and summary statistics for each phenotype. We computed the precision at various recall rates as previously described [48]. Precision is defined as the number of genes correctly classified as having a certain phenotype (true positives, TP) divided by the total number of genes classified as having that phenotype (TP and false positives, FP):
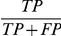



Recall is defined as the percentage of genes annotated to a given phenotype that were classified as having that phenotype:
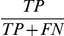
where FN represents the number of false negative predictions.

There is a trade off between precision and recall in that the most confident predictions are more likely to be accurate, whereas in order to achieve high levels of recall, we must accept a lower level of precision. As such, precision values are measured at many levels of recall to produce a curve. In order to produce single number summary statistics from these curves, we use the area under the precision-recall curve (AUPRC) as well as the precision at fixed levels of recall, including 1%, 10%, 20% and 50%.

### Ethics statement and animal husbandry

All studies and procedures were approved by the Institutional ACUC of The Jackson Laboratory. The B6.129S4-***Timp2*** *^tm1Pds^*/J (Stock Number 0008120) and B6.129-***Abcg8*** *^tm1Elk^*/J (Stock Number 008763) mice were originally purchased from the resource colonies of The Jackson Laboratory (Bar Harbor, ME) and colonies were maintained by pair mating heterozygous mice. After weaning, mice were maintained in groups of 3–5 in polycarbonate boxes (130 cm^2^) on bedding of sterilized white pine shavings under conditions of 12 hours light; 12 hours darkness. All mice used in this study had free access to water and diet for the duration of the study.

### Peripheral quantitative computed tomography assessment of volumetric BMD

Total femoral volumetric BMD (vBMD) and femoral geometry was assessed *ex vivo* by peripheral quantitative computed tomography (pQCT). Specifically, mice were killed at 16 weeks of age and femurs were isolated and fixed in 95% ethanol for 14 days. Femurs were measured for density using an SA Plus pQCT densitometer (Orthometrics, Stratec SA Plus Research Unit, White Plains, NY) as previously described [49]. Daily quality control of the SA Plus instrument's operation was checked with a manufacturer supplied phantom. The bone scans were analyzed with threshold settings to separate bone from soft tissue and to separate cortical from sub-cortical bone. Precision of the SA Plus for repeated measurements of a single femur was previously found to be 1.2–1.4%. Isolated femurs were scanned at 7 locations at 2 mm intervals, beginning 0.8 mm from the distal ends of the epiphyseal condyles. Total vBMD values were calculated by dividing the total mineral content by the total bone volume and expressed as mg/mm^3^. Periosteal circumference and cortical thickness measures were made at the exact midshaft of the femur.

## Supporting Information

Figure S1 Well defined, high level MP terms were obtained from MGI, which represent a wild sampling of phenotypes. Precisions at different levels of recall were calculated for both the summed weight method (left) and the network-based SVM method (right), where the latter shows significant improvement.
(1.35 MB TIF)

Table S1 Supporting evidence for top connectors to the candidate genes.
(0.06 MB DOC)

Table S2 Interaction weights (posteriors) of local networks surrounding Timp2 and Abcg8.
(0.07 MB DOC)

Table S3 Top 100 genes predicted for association with ‘abnormal bone mineralization’.
(0.17 MB DOC)

Text S1 Integration of diverse data for constructing a functional relationship network.
(0.07 MB DOC)

Text S2 Training SVMs on raw data as a baseline for performance evaluation.
(0.06 MB DOC)

## References

[1] Mackay TF, Stone EA, Ayroles JF (2009). The genetics of quantitative traits: challenges and prospects.. Nat Rev Genet.

[2] Wang WY, Barratt BJ, Clayton DG, Todd JA (2005). Genome-wide association studies: theoretical and practical concerns.. Nat Rev Genet.

[3] Moore JH, Asselbergs FW, Williams SM (2010). Bioinformatics challenges for genome-wide association studies.. Bioinformatics.

[4] McCarthy MI, Abecasis GR, Cardon LR, Goldstein DB, Little J (2008). Genome-wide association studies for complex traits: consensus, uncertainty and challenges.. Nat Rev Genet.

[5] Ackert-Bicknell CL, Karasik D, Li Q, Smith RV, Hsu YH (2010). Mouse BMD quantitative trait loci show improved concordance with human genome wide association loci when recalculated on a new, common mouse genetic map.. J Bone Miner Res.

[6] Manolio TA, Collins FS, Cox NJ, Goldstein DB, Hindorff LA (2009). Finding the missing heritability of complex diseases.. Nature.

[7] Rivadeneira F, Styrkarsdottir U, Estrada K, Halldorsson BV, Hsu YH (2009). Twenty bone-mineral-density loci identified by large-scale meta-analysis of genome-wide association studies.. Nat Genet.

[8] Troyanskaya OG, Dolinski K, Owen AB, Altman RB, Botstein D (2003). A Bayesian framework for combining heterogeneous data sources for gene function prediction (in Saccharomyces cerevisiae).. Proc Natl Acad Sci U S A.

[9] Myers CL, Robson D, Wible A, Hibbs MA, Chiriac C (2005). Discovery of biological networks from diverse functional genomic data.. Genome Biol.

[10] Guan Y, Myers CL, Lu R, Lemischka IR, Bult CJ (2008). A genomewide functional network for the laboratory mouse.. PLoS Comput Biol.

[11] Lee I, Lehner B, Crombie C, Wong W, Fraser AG (2008). A single gene network accurately predicts phenotypic effects of gene perturbation in Caenorhabditis elegans.. Nat Genet.

[12] Rhodes DR, Tomlins SA, Varambally S, Mahavisno V, Barrette T (2005). Probabilistic model of the human protein-protein interaction network.. Nat Biotechnol.

[13] Xia K, Dong D, Han JD (2006). IntNetDB v1.0: an integrated protein-protein interaction network database generated by a probabilistic model.. BMC Bioinformatics.

[14] Workman CT, Mak HC, McCuine S, Tagne JB, Agarwal M (2006). A systems approach to mapping DNA damage response pathways.. Science.

[15] Lee I, Date SV, Adai AT, Marcotte EM (2004). A probabilistic functional network of yeast genes.. Science.

[16] Chikina MD, Huttenhower C, Murphy CT, Troyanskaya OG (2009). Global prediction of tissue-specific gene expression and context-dependent gene networks in Caenorhabditis elegans.. PLoS Comput Biol.

[17] Lee I, Ambaru B, Thakkar P, Marcotte EM, Rhee SY (2010). Rational association of genes with traits using a genome-scale gene network for Arabidopsis thaliana.. Nat Biotechnol.

[18] Huttenhower C, Haley EM, Hibbs MA, Dumeaux V, Barrett DR (2009). Exploring the human genome with functional maps.. Genome Res.

[19] McGary KL, Lee I, Marcotte EM (2007). Broad network-based predictability of Saccharomyces cerevisiae gene loss-of-function phenotypes.. Genome Biol.

[20] Linghu B, Snitkin ES, Hu Z, Xia Y, Delisi C (2009). Genome-wide prioritization of disease genes and identification of disease-disease associations from an integrated human functional linkage network.. Genome Biol.

[21] Brem, Storey, Whittle J, Kruglyak (2005). Genetic interactions between polymorphisms that affect gene expression in yeast.. Nature.

[22] Carlborg O, De Koning DJ, Manly KF, Chesler, Williams RW (2005). Methodological aspects of the genetic dissection of gene expression.. Bioinformatics (Oxford, England).

[23] Cookson W, Liang L, Abecasis G, Moffatt M, Lathrop M (2009). Mapping complex disease traits with global gene expression.. Nat Rev Genet.

[24] Alfarano C, Andrade CE, Anthony K, Bahroos N, Bajec M (2005). The Biomolecular Interaction Network Database and related tools 2005 update.. Nucleic Acids Res.

[25] Breitkreutz BJ, Stark C, Tyers M (2003). The GRID: the General Repository for Interaction Datasets.. Genome Biol.

[26] Salwinski L, Miller CS, Smith AJ, Pettit FK, Bowie JU (2004). The Database of Interacting Proteins: 2004 update.. Nucleic Acids Res.

[27] Brown KR, Jurisica I (2005). Online predicted human interaction database.. Bioinformatics.

[28] Durinck S, Moreau Y, Kasprzyk A, Davis S, De Moor B (2005). BioMart and Bioconductor: a powerful link between biological databases and microarray data analysis.. Bioinformatics.

[29] O'Brien KP, Remm M, Sonnhammer EL (2005). Inparanoid: a comprehensive database of eukaryotic orthologs.. Nucleic Acids Res.

[30] Siddiqui AS, Khattra J, Delaney AD, Zhao Y, Astell C (2005). A mouse atlas of gene expression: large-scale digital gene-expression profiles from precisely defined developing C57BL/6J mouse tissues and cells.. Proc Natl Acad Sci U S A.

[31] Su AI, Wiltshire T, Batalov S, Lapp H, Ching KA (2004). A gene atlas of the mouse and human protein-encoding transcriptomes.. Proc Natl Acad Sci U S A.

[32] Zhang W, Morris QD, Chang R, Shai O, Bakowski MA (2004). The functional landscape of mouse gene expression.. J Biol.

[33] Bult CJ, Eppig JT, Kadin JA, Richardson JE, Blake JA (2008). The Mouse Genome Database (MGD): mouse biology and model systems.. Nucleic Acids Res.

[34] Ashburner M, Ball CA, Blake JA, Botstein D, Butler H (2000). Gene ontology: tool for the unification of biology. The Gene Ontology Consortium.. Nat Genet.

[35] Smith CL, Goldsmith CA, Eppig JT (2005). The Mammalian Phenotype Ontology as a tool for annotating, analyzing and comparing phenotypic information.. Genome Biol.

[36] Vapnik VN (2000). The nature of statistical learning theory.

[37] Eppig JT, Bult CJ, Kadin JA, Richardson JE, Blake JA (2005). The Mouse Genome Database (MGD): from genes to mice–a community resource for mouse biology.. Nucleic Acids Res.

[38] Guan Y, Myers CL, Hess DC, Barutcuoglu Z, Caudy AA (2008). Predicting gene function in a hierarchical context with an ensemble of classifiers.. Genome Biol.

[39] Wergedal JE, Ackert-Bicknell CL, Tsaih SW, Sheng MH, Li R (2006). Femur mechanical properties in the F2 progeny of an NZB/B1NJ×RF/J cross are regulated predominantly by genetic loci that regulate bone geometry.. J Bone Miner Res.

[40] Varghese S (2006). Matrix metalloproteinases and their inhibitors in bone: an overview of regulation and functions.. Front Biosci.

[41] Hatori K, Sasano Y, Takahashi I, Kamakura S, Kagayama M (2004). Osteoblasts and osteocytes express MMP2 and -8 and TIMP1, -2, and -3 along with extracellular matrix molecules during appositional bone formation.. Anat Rec A Discov Mol Cell Evol Biol.

[42] Lazary A, Kosa JP, Tobias B, Lazary J, Balla B (2008). Single nucleotide polymorphisms in new candidate genes are associated with bone mineral density and fracture risk.. Eur J Endocrinol.

[43] Tarr PT, Tarling EJ, Bojanic DD, Edwards PA, Baldan A (2009). Emerging new paradigms for ABCG transporters.. Biochim Biophys Acta.

[44] Huang MS, Lu J, Ivanov Y, Sage AP, Tseng W (2008). Hyperlipidemia impairs osteoanabolic effects of PTH.. J Bone Miner Res.

[45] Gerdes LU, Vestergaard P, Hermann AP, Mosekilde L (2001). Regional and hormone-dependent effects of apolipoprotein E genotype on changes in bone mineral in perimenopausal women.. J Bone Miner Res.

[46] Taes Y, Lapauw B, G V, De Bacquer D, Goemaere S (2010). Prevalent fractures are related to cortical bone geometry in young healthy men at age of peak bone mass.. J Bone Miner Res.

[47] Fu WJ, Carroll RJ, Wang S (2005). Estimating misclassification error with small samples via bootstrap cross-validation.. Bioinformatics.

[48] Jesse D, Mark G (2006). The relationship between Precision-Recall and ROC curves..

[49] Ackert-Bicknell CL, Shockley KR, Horton LG, Lecka-Czernik B, Churchill GA (2009). Strain-specific effects of rosiglitazone on bone mass, body composition, and serum insulin-like growth factor-I.. Endocrinology.

[50] Hu Z, Snitkin ES, DeLisi C (2008). VisANT: an integrative framework for networks in systems biology.. Brief Bioinform.

