# Supplementary figures and images for: Functional Genomics Complements Quantitative Genetics in Identifying Disease-Gene Associations
Source: PLoS Comput Biol. 2010 Nov 11;6(11):e1000991. doi: 10.1371/journal.pcbi.1000991 (PMC2978695; doi:10.1371/journal.pcbi.1000991)

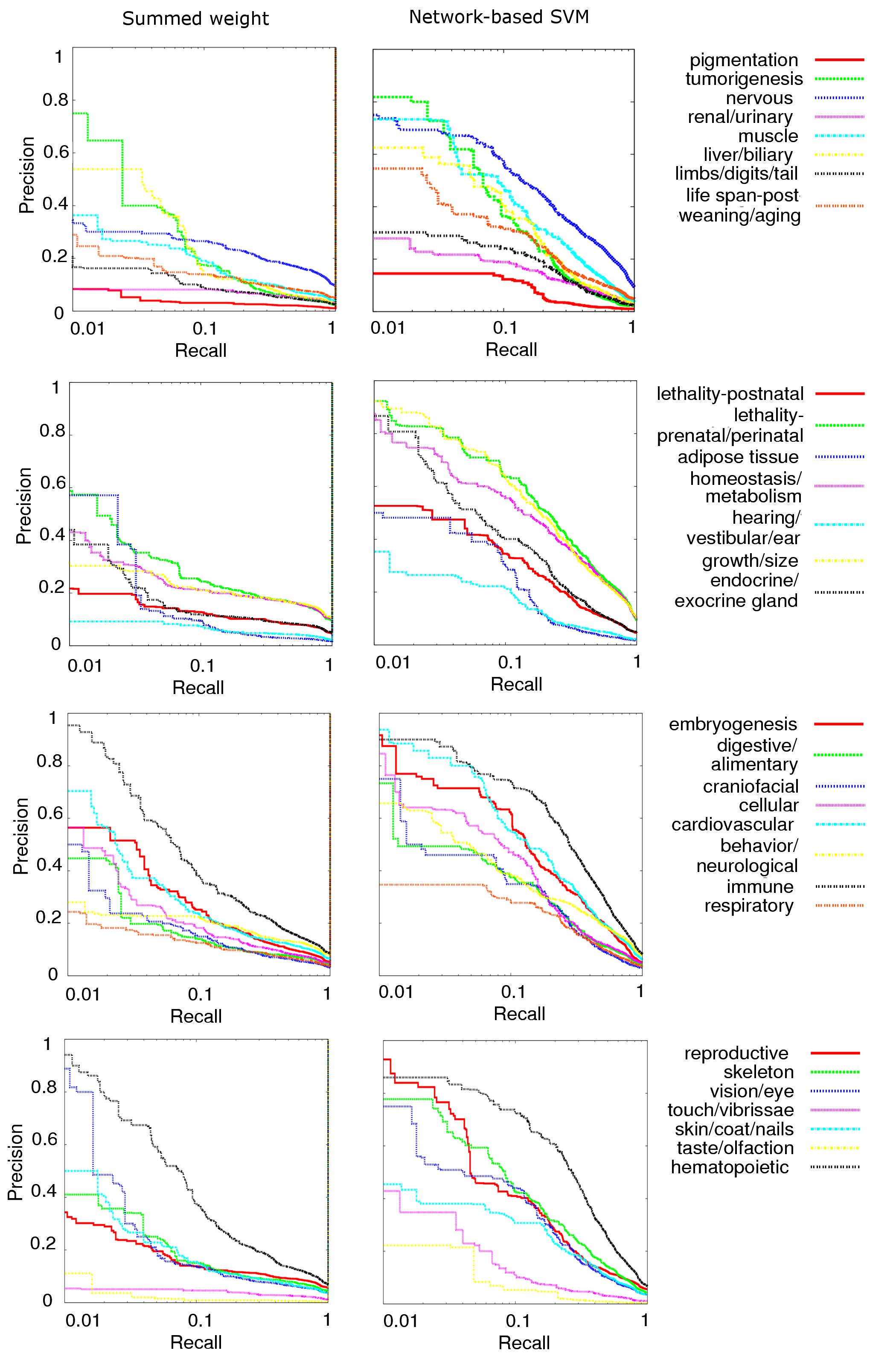

Supplement: Figure S1 — Well defined, high level MP terms were obtained from MGI, which represent a wild sampling of phenotypes. Precisions at different levels of recall were calculated for both the summed weight method (left) and the network-based SVM method (right), where the latter shows significant improvement. (1.35 MB TIF) [file pcbi.1000991.s001.tif]
