# Supplementary material for: Functional Genomics Complements Quantitative Genetics in Identifying Disease-Gene Associations
Source: PLoS Comput Biol. 2010 Nov 11;6(11):e1000991. doi: 10.1371/journal.pcbi.1000991 (PMC2978695; doi:10.1371/journal.pcbi.1000991)
Supplement: Table S1 — Supporting evidence for top connectors to the candidate genes. (0.06 MB DOC) [file pcbi.1000991.s002.doc]

**Supplementary Table S**1. Supporting evidence for top connectors to the candidate genes.

| Functionally related genes to TIMP2 | Support evidence type | Supporting data | Raw value (as measurement of similarity) | Interpreted as (single dataset posterior) |
| --- | --- | --- | --- | --- |
| MMP8 | Physical interactions from homology | OPHID | 1 | 0.26 |
|  | Phylogenetic profiles | BioMart | 0.36 | 0.029 |
|  | Expression | Zhang et al. | 0.68 | 0.03 |
| MMP2 | Physical interaction from homology | OPHID | 1 | 0.26 |
|  | Phylogenetic profiles | BioMart | 0.61 | 0.031 |
|  | Expression | SAGE | 6.72 | 0.066 |
|  | Expression | Su et al. | 1.34 | 0.035 |
| MMP14 | Phylogenetic profiles | InParanoid | 0.76 | 0.035 |
|  | Physical interactions from homology | OPHID | 1 | 0.26 |
|  | Phylogenetic profiles | BioMart | 2.25 | 0.032 |
|  | Expression | SAGE | 2.12 | 0.035 |
| SPARC | Phylogenetic profiles | InParanoid | 1.93 | 0.038 |
|  | Expression | SAGE | 6.80 | 0.067 |
|  | Expression | Su et al. | 1.94 | 0.043 |
| OSBPL9 | Phylogenetic profiles | InParanoid | 0.92 | 0.035 |
|  | Expression | SAGE | 4.62 | 0.052 |
|  | Expression | Zhang et al. | 1.77 | 0.03 |
| Functionally related genes to ABCG8 | Support evidence type | Supporting data | Raw value (as measurement of similarity) | Interpreted as (single dataset posterior) |
| ABCG5 | Phylogenetic profiles | InParanoid | 1.47 | 0.035 |
|  | Physical interactions from homology | OPHID | 1 | 0.26 |
|  | Phylogenetic profiles | BioMart | 1.56 | 0.032 |
|  | Expression | Su et al. | 6.78 | 0.085 |
| FXYD6 | Phylogenetic profiles | BioMart | 0.29 | 0.029 |
|  | Expression | SAGE | 0.67 | 0.031 |
|  | Expression | Zhang et al. | 8.86 | 0.12 |
| BC034204 | Phylogenetic profiles | InParanoid | 2.14 | 0.039 |
|  | Phylogenetic profiles | BioMart | 2.49 | 0.032 |
|  | Expression | Zhang et al. | 6.41 | 0.12 |
| COL1A2 | Phylogenetic profiles | InParanoid | 2.37 | 0.039 |
|  | Phylogenetic profiles | BioMart | 2.70 | 0.036 |
|  | Expression | Zhang et al. | 6.43 | 0.12 |
| COL1A1 | Phylogenetic profiles | InParanoid | 1.52 | 0.039 |
|  | Phylogenetic profiles | BioMart | 1.46 | 0.031 |
|  | Expression | SAGE | 0.29 | 0.028 |
|  | Expression | Zhang et al. | 5.85 | 0.12 |
