# Supplementary material for: Functional Genomics Complements Quantitative Genetics in Identifying Disease-Gene Associations
Source: PLoS Comput Biol. 2010 Nov 11;6(11):e1000991. doi: 10.1371/journal.pcbi.1000991 (PMC2978695; doi:10.1371/journal.pcbi.1000991)
Supplement: Table S2 — Interaction weights (posteriors) of local networks surrounding Timp2 and Abcg8. (0.07 MB DOC) [file pcbi.1000991.s003.doc]

**Table S2. Interaction weights (posteriors) of local networks surrounding *Timp2* and *Abcg8*. The shared interaction partners between *Timp2* and *Abcg8* are highlighted with grey background.**

| Timp2 | | | Abcg8 | | |
| --- | --- | --- | --- | --- | --- |
| First level interactions | | | | | |
| Timp2 | Mmp2 | 0.292005 | Abcg8 | Abcg5 | 0.389284 |
| Timp2 | Mmp14 | 0.284632 | Abcg8 | Col1a2 | 0.144639 |
| Timp2 | Mmp8 | 0.165517 | Abcg8 | BC034204 | 0.135686 |
| Timp2 | Sparc | 0.064598 | Abcg8 | Col1a1 | 0.123176 |
| Timp2 | Osbpl9 | 0.061465 | Abcg8 | Fxyd6 | 0.12154 |
| Second level interactions | | | | | |
| Mmp2 | Col1a1 | 0.448196 | Abcg5 | Ace2 | 0.110012 |
| Mmp2 | Cldn1 | 0.330733 | Abcg5 | Rbp2 | 0.102599 |
| Mmp2 | Bace1 | 0.324228 | Abcg5 | Tm4sf5 | 0.102264 |
| Mmp2 | Thbs1 | 0.30762 | Abcg5 | Creb3l3 | 0.095004 |
| Mmp14 | Timp3 | 0.269482 | Col1a2 | Sparc | 0.473243 |
| Mmp14 | Cldn1 | 0.2583 | Col1a2 | Lum | 0.471044 |
| Mmp14 | Lrp1 | 0.211945 | Col1a2 | Bgn | 0.426064 |
| Mmp14 | Itgb8 | 0.20589 | Col1a2 | Col1a1 | 0.39329 |
| Mmp8 | Ms4a3 | 0.32286 | Col1a2 | Fn1 | 0.366115 |
| Mmp8 | Trem3 | 0.31396 | BC034204 | Fmod | 0.149959 |
| Mmp8 | Ifitm6 | 0.287058 | BC034204 | Abcg8 | 0.135686 |
| Mmp8 | Mpo | 0.26481 | BC034204 | Cdh17 | 0.125844 |
| Mmp8 | Ngp | 0.251735 | BC034204 | Lum | 0.100187 |
| Sparc | Col1a2 | 0.473243 | BC034204 | Col1a2 | 0.09644 |
| Sparc | Col1a1 | 0.427408 | Col1a1 | Mmp2 | 0.448196 |
| Sparc | Plat | 0.308866 | Col1a1 | Sparc | 0.427408 |
| Sparc | Sdc2 | 0.301052 | Col1a1 | Bgn | 0.371275 |
| Sparc | Thbs1 | 0.300142 | Col1a1 | Fn1 | 0.36217 |
| Osbpl9 | Osbpl11 | 0.368972 | Fxyd6 | Cep27 | 0.237289 |
| Osbpl9 | Osbpl`0 | 0.25167 | Fxyd6 | Trp53 | 0.222257 |
| Osbpl9 | Osbpl2 | 0.152867 | Fxyd6 | C030048B08Rik | 0.208928 |
| Osbpl9 | Osbpl1a | 0.138046 | Fxyd6 | Trim27 | 0.202349 |
| Osbp9 | Osbpl3 | 0.127123 | Fxyd6 | Bcar1 | 0.185786 |
