# Supplementary material for: Functional Genomics Complements Quantitative Genetics in Identifying Disease-Gene Associations
Source: PLoS Comput Biol. 2010 Nov 11;6(11):e1000991. doi: 10.1371/journal.pcbi.1000991 (PMC2978695; doi:10.1371/journal.pcbi.1000991)
Supplement: Table S3 — Top 100 genes predicted for association with ‘abnormal bone mineralization’. (0.17 MB DOC) [file pcbi.1000991.s004.doc]

**Table S3.** Top 100 genes predicted for association with ‘abnormal bone mineralization’ (MP:0002869). For each of the top 100 predictions, we show the raw SVM score, the normalized SVM probability, whether the gene was previously associated with BMD (“Prev Known?”), whether the gene lies underneath a BMD QTL (“QTL Candidate?”), and whether a live knockout was available at the time of the study (“Live KO?”). *Timp2* and *Abcg8* are the two highest ranking genes that meet our criteria of 1) no previous association with BMD, 2) not a candidate from QTL or GWAS, and 3) with an existing, live knockout strain available.

*Note that *Abcg8* lies underneath a QTL peak, but is not a candidate since there are no polymorphisms in Abcg8 between the strains crossed in that study.

| ***Rank*** | ***MGI ID*** | ***Symbol*** | ***SVM Score*** | ***SVM Probability*** | ***Prev Known?*** | ***QTL Candidate?*** | ***Live KO?*** | ***Study Candidate*** |
| --- | --- | --- | --- | --- | --- | --- | --- | --- |
| 1 | MGI:104769 | FKBP10 | 0.142857143 | 1 | 0 | 1 | 0 | 0 |
| 2 | MGI:98373 | SPARC | 0.142857143 | 1 | 0 | 1 | 0 | 0 |
| 3 | MGI:3036234 | HDAC4 | 0.142857143 | 1 | 0 | 1 | 0 | 0 |
| 4 | MGI:1338035 | ITGB8 | 0.142857143 | 1 | 0 | 1 | 0 | 0 |
| 5 | MGI:88467 | COL1A1 | 0.142857143 | 1 | 1 | 1 | 0 | 0 |
| 6 | MGI:99918 | MECP2 | 0.142857143 | 1 | 0 | 1 | 1 | 0 |
| 7 | MGI:101927 | DLX6 | 0.142857143 | 1 | 0 | 1 | 0 | 0 |
| 8 | MGI:1314872 | HIPK2 | 0.142857143 | 1 | 0 | 1 | 0 | 0 |
| 9 | MGI:97874 | RB1 | 0.142857143 | 1 | 0 | 1 | 0 | 0 |
| 10 | MGI:97169 | MSX2 | 0.142857143 | 1 | 0 | 1 | 0 | 0 |
| 11 | MGI:97342 | NID1 | 0.142857143 | 1 | 0 | 1 | 0 | 0 |
| **12** | **MGI:98753** | ***TIMP2*** | **0.142857143** | **1** | **0** | **0** | **1** | **1** |
| 13 | MGI:95832 | GRN | 0.142857143 | 1 | 0 | 1 | 0 | 0 |
| 14 | MGI:88158 | BGN | 0.142857143 | 1 | 0 | 1 | 0 | 0 |
| 15 | MGI:95488 | FBLN2 | 0.142857143 | 1 | 0 | 1 | 0 | 0 |
| 16 | MGI:95574 | FOS | 0.142857143 | 1 | 0 | 1 | 0 | 0 |
| 17 | MGI:88059 | APP | 0.142857143 | 1 | 0 | 0 | 0 | 0 |
| 18 | MGI:97009 | MMP2 | 0.142857143 | 1 | 0 | 1 | 0 | 0 |
| 19 | MGI:99851 | CBFB | 0.142857143 | 1 | 0 | 1 | 0 | 0 |
| 20 | MGI:107489 | PHEX | 0.142857143 | 1 | 1 | 0 | 1 | 0 |
| 21 | MGI:88468 | COL1A2 | 0.142857143 | 1 | 1 | 0 | 1 | 0 |
| 22 | MGI:96600 | ITGA2 | 0.136363636 | 1 | 0 | 1 | 0 | 0 |
| 23 | MGI:94910 | DMP1 | 0.133333333 | 1 | 0 | 1 | 0 | 0 |
| 24 | MGI:98754 | TIMP3 | 0.133333333 | 1 | 0 | 1 | 0 | 0 |
| 25 | MGI:95487 | FBLN1 | 0.133333333 | 1 | 0 | 1 | 0 | 0 |
| 26 | MGI:96817 | LOX | 0.133333333 | 1 | 0 | 1 | 0 | 0 |
| **27** | **MGI:1914720** | ***ABCG8*** | **0.133333333** | **0.999999999** | **0** | ***0** | **1** | **1** |
| 28 | MGI:1346833 | NR5A1 | 0.133333333 | 0.999999984 | 0 | 1 | 0 | 0 |
| 29 | MGI:1860118 | SEMA3D | 0.133333333 | 0.999999981 | 0 | 1 | 0 | 0 |
| 30 | MGI:101900 | MMP14 | 0.133333333 | 0.999999974 | 1 | 1 | 0 | 0 |
| 31 | MGI:107569 | EPX | 0.129032258 | 0.999999836 | 0 | 1 | 0 | 0 |
| 32 | MGI:99217 | CDH11 | 0.125 | 0.999999747 | 0 | 1 | 0 | 0 |
| 33 | MGI:1201674 | SMAD3 | 0.121212121 | 0.999999672 | 0 | 1 | 0 | 0 |
| 34 | MGI:1277201 | COL13A1 | 0.117647059 | 0.999999141 | 0 | 1 | 0 | 0 |
| 35 | MGI:107899 | CD36 | 0.114285714 | 0.999997859 | 0 | 1 | 0 | 0 |
| 36 | MGI:96607 | ITGAM | 0.111111111 | 0.999992275 | 0 | 1 | 1 | 0 |
| 37 | MGI:88446 | COL11A1 | 0.108108108 | 0.99995956 | 0 | 1 | 0 | 0 |
| 38 | MGI:108051 | SMAD2 | 0.105263158 | 0.999955484 | 0 | 1 | 0 | 0 |
| 39 | MGI:1336879 | FKBP7 | 0.102564103 | 0.999953441 | 0 | 1 | 0 | 0 |
| 40 | MGI:2444418 | SCARA3 | 0.1 | 0.999934384 | 0 | 1 | 0 | 0 |
| 41 | MGI:1926189 | EMILIN1 | 0.097560976 | 0.99990861 | 0 | 1 | 0 | 0 |
| 42 | MGI:1202395 | MMP8 | 0.095238095 | 0.999738353 | 0 | 0 | 0 | 0 |
| 43 | MGI:102793 | FSTL1 | 0.093023256 | 0.999596881 | 0 | 1 | 0 | 0 |
| 44 | MGI:1916788 | ANTXR1 | 0.090909091 | 0.99943672 | 0 | 1 | 0 | 0 |
| 45 | MGI:108075 | SMO | 0.088888889 | 0.999370499 | 0 | 1 | 1 | 0 |
| 46 | MGI:103294 | PRG2 | 0.086956522 | 0.999332664 | 0 | 1 | 0 | 0 |
| 47 | MGI:105085 | RBL2 | 0.085106383 | 0.999063605 | 0 | 1 | 0 | 0 |
| 48 | MGI:109347 | LUM | 0.083333333 | 0.99895249 | 0 | 1 | 0 | 0 |
| 49 | MGI:99892 | LAMA1 | 0.081632653 | 0.99849634 | 0 | 1 | 0 | 0 |
| 50 | MGI:88457 | COL5A1 | 0.08 | 0.9983913 | 0 | 1 | 0 | 0 |
| 51 | MGI:1918881 | PANX3 | 0.078431373 | 0.998268448 | 0 | 1 | 0 | 0 |
| 52 | MGI:107818 | MRC2 | 0.076923077 | 0.998172134 | 0 | 1 | 0 | 0 |
| 53 | MGI:108295 | PXN | 0.075471698 | 0.998079152 | 0 | 1 | 0 | 0 |
| 54 | MGI:96438 | IGFBP3 | 0.075 | 0.998028301 | 0 | 1 | 0 | 0 |
| 55 | MGI:2151110 | PRELP | 0.075 | 0.997302518 | 0 | 1 | 0 | 0 |
| 56 | MGI:97528 | PDGFB | 0.075 | 0.993750583 | 0 | 1 | 0 | 0 |
| 57 | MGI:1095396 | COL16A1 | 0.075 | 0.993674575 | 0 | 1 | 0 | 0 |
| 58 | MGI:1914877 | OLFML3 | 0.075 | 0.993235012 | 0 | 1 | 0 | 0 |
| 59 | MGI:1924268 | COL25A1 | 0.075 | 0.993020529 | 0 | 1 | 0 | 0 |
| 60 | MGI:1923797 | TMEM101 | 0.075 | 0.990297098 | 0 | 1 | 0 | 0 |
| 61 | MGI:99959 | TGFBI | 0.075 | 0.989636066 | 0 | 1 | 0 | 0 |
| 62 | MGI:96608 | ITGAV | 0.075 | 0.988773372 | 0 | 1 | 0 | 0 |
| 63 | MGI:94872 | DCN | 0.075 | 0.985824451 | 0 | 1 | 0 | 0 |
| 64 | MGI:2444926 | CKAP4 | 0.075 | 0.985419284 | 0 | 1 | 0 | 0 |
| 65 | MGI:1341847 | HHIP | 0.075 | 0.968853931 | 0 | 1 | 0 | 0 |
| 66 | MGI:107532 | KPNB1 | 0.075 | 0.967821219 | 0 | 1 | 0 | 0 |
| 67 | MGI:96604 | ITGA5 | 0.075 | 0.966492814 | 0 | 0 | 0 | 0 |
| 68 | MGI:1276535 | NCOA3 | 0.075 | 0.965505162 | 0 | 1 | 0 | 0 |
| 69 | MGI:1891427 | FGF23 | 0.075 | 0.963288699 | 1 | 1 | 0 | 0 |
| 70 | MGI:88039 | APC | 0.075 | 0.956078217 | 0 | 1 | 1 | 0 |
| 71 | MGI:2429611 | NLRX1 | 0.075 | 0.940543914 | 0 | 1 | 0 | 0 |
| 72 | MGI:1919959 | C1QTNF6 | 0.075 | 0.932664217 | 0 | 1 | 0 | 0 |
| 73 | MGI:95556 | FLNA | 0.075 | 0.923724347 | 0 | 1 | 0 | 0 |
| 74 | MGI:1351602 | DGUOK | 0.075 | 0.921204804 | 0 | 1 | 0 | 0 |
| 75 | MGI:97856 | RARA | 0.075 | 0.910658842 | 0 | 1 | 0 | 0 |
| 76 | MGI:1891209 | EFEMP2 | 0.075 | 0.893567887 | 0 | 1 | 0 | 0 |
| 77 | MGI:106613 | DVL2 | 0.075 | 0.886631578 | 0 | 1 | 1 | 0 |
| 78 | MGI:106925 | CLDN11 | 0.075 | 0.880070867 | 0 | 1 | 0 | 0 |
| 79 | MGI:96646 | JUN | 0.075 | 0.827448516 | 0 | 1 | 0 | 0 |
| 80 | MGI:101926 | DLX5 | 0.075 | 0.818957795 | 1 | 1 | 0 | 0 |
| 81 | MGI:2670964 | FAM46A | 0.074074074 | 0.7607272 | 0 | 1 | 0 | 0 |
| 82 | MGI:1309472 | LTB4R1 | 0.073170732 | 0.759235827 | 0 | 1 | 1 | 0 |
| 83 | MGI:101835 | TRAF2 | 0.072289157 | 0.74529336 | 0 | 1 | 0 | 0 |
| 84 | MGI:1096363 | GP5 | 0.071428571 | 0.679872557 | 0 | 1 | 0 | 0 |
| 85 | MGI:1928098 | TSPAN3 | 0.070588235 | 0.655047149 | 0 | 1 | 0 | 0 |
| 86 | MGI:88448 | COL12A1 | 0.069767442 | 0.650967186 | 0 | 1 | 0 | 0 |
| 87 | MGI:2158468 | MS4A3 | 0.068965517 | 0.596972624 | 0 | 1 | 0 | 0 |
| 88 | MGI:1347064 | MED21 | 0.068181818 | 0.587815295 | 0 | 0 | 0 | 0 |
| 89 | MGI:1194505 | C1QBP | 0.06741573 | 0.537436458 | 0 | 1 | 0 | 0 |
| 90 | MGI:1860512 | PRPF40A | 0.066666667 | 0.510405656 | 0 | 1 | 0 | 0 |
| 91 | MGI:1340026 | MMP13 | 0.065934066 | 0.465824993 | 0 | 0 | 0 | 0 |
| 92 | MGI:893580 | PRTN3 | 0.065217391 | 0.455530627 | 0 | 1 | 0 | 0 |
| 93 | MGI:88447 | COL11A2 | 0.064516129 | 0.450839223 | 0 | 1 | 0 | 0 |
| 94 | MGI:1859631 | PDGFC | 0.063829787 | 0.447096072 | 0 | 1 | 0 | 0 |
| 95 | MGI:1889800 | MYOT | 0.063157895 | 0.430026593 | 0 | 1 | 0 | 0 |
| 96 | MGI:1197008 | WISP1 | 0.0625 | 0.427891285 | 0 | 1 | 0 | 0 |
| 97 | MGI:1915838 | CTHRC1 | 0.06185567 | 0.427874124 | 0 | 1 | 0 | 0 |
| 98 | MGI:88180 | BMP4 | 0.06122449 | 0.40457787 | 0 | 1 | 0 | 0 |
| 99 | MGI:98216 | RXRG | 0.060606061 | 0.395888255 | 0 | 1 | 0 | 0 |
| 100 | MGI:1278319 | FPR2 | 0.06 | 0.382224476 | 0 | 1 | 0 | 0 |
