# Supplementary material for: Functional Genomics Complements Quantitative Genetics in Identifying Disease-Gene Associations
Source: PLoS Comput Biol. 2010 Nov 11;6(11):e1000991. doi: 10.1371/journal.pcbi.1000991 (PMC2978695; doi:10.1371/journal.pcbi.1000991)
Supplement: Text S1 — Integration of diverse data for constructing a functional relationship network. (0.07 MB DOC) [file pcbi.1000991.s005.doc]

**Supplemental Text S1**

**Integration of diverse data for constructing a functional relationship network**

We gathered diverse sources of data to construct a functional relationship network for the laboratory mouse following the computational protocol developed in [1]. In general, this framework allows integration of all types of functional genomic data, regardless of individual reliability and source. In this study, we intended to examine the computational value of such networks in predicting geneotype-phenotype associations. Therefore, we must exclude genome-wide phenotypic data to avoid circularity. As such, the data included in our functional relationship network include protein-protein physical interactions [2-5], phylogenetic profiles [6,7], homologous functional relationship predictions in other model organisms [8], expression data and tissue localization data [9-11].

In the functional relationship network, each gene is represented as a node and the connection between each pair of genes represents the probability that the two genes are functionally related (i.e. participate in the same biological process). To derive this probability, we require a gold standard set of functionally related pairs of genes and pair-wise high throughput data suitable for integration.

First, we created a functional gold standard derived from the Gene Ontology (GO). Positive (functionally related) pairs were defined by co-annotation to GO biological process (BP) terms that are sufficiently specific (e.g. terms with less than 300 hundred genes annotated). This GO size limitation is to ensure that very broad terms representing non-specific biological processes are not included, such as “regulation” or “cellular process.” Negative (unrelated) pairs were defined as pairs where both members have at least one specific GO BP term annotation, yet they are not co-annotated to any specific term. These positive and negative pairs together construct the gold standard set which is later used for integrating the posteriors derived from all datasets.

Second, we transformed each input dataset into gene-gene pair-wise values that represent the similarity between genes. These values were later used to estimate the accuracy of each dataset. Each dataset was transformed in a manner suitable for that data type:

1. Protein-protein physical interaction data:
   For each dataset, an existing protein-protein interacting pair has a pair-wise score of 1. All other protein pairs have a score of 0. For BIND [2], we considered all the inter-connections between the targets of a certain bait to be positive pairs, with a score of 1. For OPHID [5], all unique physical interactions were mapped to mouse using InParanoid [7].
2. Homologous functional relationship predictions:
   Based on our previous analyses, the fact that a pair of proteins participate in the same biological process in one species is highly suggestive that these proteins will also be functionally related in an orthologous species [1]. *S. cerevisiae* is the most well-understood model organism where a large fraction of genes have annotations. We therefore homologously mapped the functional relationship prediction scores from yeast [8] to mouse as a source of functional genomic data.
3. Expression and Tissue localization datasets:
   For each expression dataset, we calculated the Pearson correlation coefficient to quantify the levels of co-expression between each pair of genes. This Pearson correlation coefficient was later Fisher’s *z*-transformed and then normally transformed to ensure comparable, normal distribution between datasets.
4. Homology data:
   For homology data, we counted the number of organisms in which a pair of proteins co-occurs. However, based on our previous analysis, these data are negligible in our integration results [1].

Once we obtained our gold standard and pair-wise input datasets, we applied a Bayesian network to integrate these datasets and predict protein functional relationships between each pair of proteins. The probability of a specific pair being functionally related is given by [1]:

$$P(FR|E_{1},E_{2},...E_{n})=\frac{1}{Z}P(FR)\prod_{i=1}^{n} p(E_{i}|FR)$$

where *FR* represents the predicted probability of functional relationship, *E_i_* represents the score of the pair for each dataset (1 to *n*), and *Z* is a normalization factor.

*References:*

1. Guan Y, Myers CL, Lu R, Lemischka IR, Bult CJ, et al. (2008) A genomewide functional network for the laboratory mouse. PLoS Comput Biol 4: e1000165.

2. Alfarano C, Andrade CE, Anthony K, Bahroos N, Bajec M, et al. (2005) The Biomolecular Interaction Network Database and related tools 2005 update. Nucleic Acids Res 33: D418-424.

3. Breitkreutz BJ, Stark C, Tyers M (2003) The GRID: the General Repository for Interaction Datasets. Genome Biol 4: R23.

4. Salwinski L, Miller CS, Smith AJ, Pettit FK, Bowie JU, et al. (2004) The Database of Interacting Proteins: 2004 update. Nucleic Acids Res 32: D449-451.

5. Brown KR, Jurisica I (2005) Online predicted human interaction database. Bioinformatics 21: 2076-2082.

6. Durinck S, Moreau Y, Kasprzyk A, Davis S, De Moor B, et al. (2005) BioMart and Bioconductor: a powerful link between biological databases and microarray data analysis. Bioinformatics 21: 3439-3440.

7. O'Brien KP, Remm M, Sonnhammer EL (2005) Inparanoid: a comprehensive database of eukaryotic orthologs. Nucleic Acids Res 33: D476-480.

8. Myers CL, Robson D, Wible A, Hibbs MA, Chiriac C, et al. (2005) Discovery of biological networks from diverse functional genomic data. Genome Biol 6: R114.

9. Siddiqui AS, Khattra J, Delaney AD, Zhao Y, Astell C, et al. (2005) A mouse atlas of gene expression: large-scale digital gene-expression profiles from precisely defined developing C57BL/6J mouse tissues and cells. Proc Natl Acad Sci U S A 102: 18485-18490.

10. Su AI, Wiltshire T, Batalov S, Lapp H, Ching KA, et al. (2004) A gene atlas of the mouse and human protein-encoding transcriptomes. Proc Natl Acad Sci U S A 101: 6062-6067.

11. Zhang W, Morris QD, Chang R, Shai O, Bakowski MA, et al. (2004) The functional landscape of mouse gene expression. J Biol 3: 21.
