# Supplementary material for: Functional Genomics Complements Quantitative Genetics in Identifying Disease-Gene Associations
Source: PLoS Comput Biol. 2010 Nov 11;6(11):e1000991. doi: 10.1371/journal.pcbi.1000991 (PMC2978695; doi:10.1371/journal.pcbi.1000991)
Supplement: Text S2 — Training SVMs on raw data as a baseline for performance evaluation. (0.06 MB DOC) [file pcbi.1000991.s006.doc]

**Supplemental Text S2**

**Training SVMs on raw data as a baseline for performance evaluation**

The main algorithm developed in this paper utilizes a functional relationship network as input to an SVM that predicts gene-phenotype associations as a classification problem. Specifically, we constructed a feature space consisting of the network connection weights to all positive examples of a phenotype as feature vectors.

An intuitive alternative to this approach is to use all of the original data that was integrated into our functional relationship network as the feature space, and to train SVMs using these features [1]. Our raw data consisted of 13048 features derived from the data sources discussed in Supplemental Text S1. This large number of features presents two major problems for SVM classification. First, the time used for learning a SVM is *O(nN^2^)*, where *n* is the number of examples and *N* is the number of features. Therefore training the raw data based SVM is approximately 2000 to 200,000 more time-consuming than using our network-based approach. Nevertheless, we tested the performance of raw SVM on predicting the high-level phenotypic terms and compared it to the network-based SVM approach. Second, the larger feature space of the raw data-based SVM is not well-suited for our case where the number of training examples is much smaller than the number of features. Since the number of training examples available to us is limited, the raw SVM is more likely to overfit or lose generalizability. As such, the raw SVM performed significantly worse than our network-based SVM approach in all but one case, most likely due to this ‘curse of dimensionality’ as shown below.


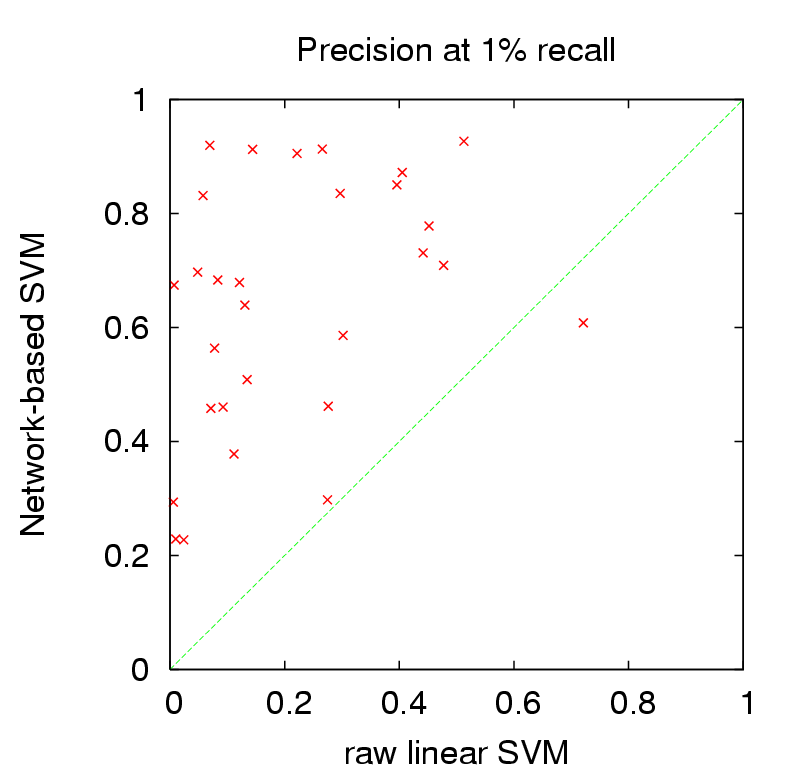


1. Guan Y, Myers CL, Hess DC, Barutcuoglu Z, Caudy AA, et al. (2008) Predicting gene function in a hierarchical context with an ensemble of classifiers. Genome Biol 9 Suppl 1: S3.
